# Microfluidic Wound-Healing Assay for Comparative Study on Fluid Dynamic, Chemical and Mechanical Wounding on Microglia BV2 Migration

**DOI:** 10.3390/mi15081004

**Published:** 2024-08-02

**Authors:** Ehsan Yazdanpanah Moghadam, Nahum Sonenberg, Muthukumaran Packirisamy

**Affiliations:** 1 Optical-Bio Microsystems Laboratory, Micro-Nano-Bio Integration Center, Department of Mechanical and Industrial Engineering, Concordia University, Montreal, QC H3G 1M8, Canada; e.yazdan.m@gmail.com; 2 Department of Biochemistry, Goodman Cancer Research Center, McGill University, Montreal, QC H3G 1Y62, Canada; nahum.sonenberg@mcgill.ca

**Keywords:** microfluidic cell adhesion assay, microfluidic migration assay, chemical and mechanical wound-healing assays, microglia BV2 cells, fluid loading

## Abstract

Microglial cells, or brain immune cells, are highly dynamic and continuously migrate in pathophysiological conditions. Their adhesion, as a physical characteristic, plays a key role in migration. In this study, we presented a microfluidic chip combination of two assays: a microglial BV2 adhesion assay and a wound-healing migration assay. The chip could create the cell-free area (wound) under chemical stimuli with trypsin (chemical assay) and also mechanical stimuli with the PBS flow (mechanical assay). The microfluidic chip functioned as the cell adhesion assay during wounding, when the cell adhesion of microglia BV2 cells was characterized by the cell removal time under various shear stress ranges. The cell detachment pattern on the glass substrate was found under physiological conditions. After wounding, the chip operated as a migration assay; it was shown that cell migration in the cell-free area generated chemically with trypsin was highly improved compared to mechanical cell-free area creations with PBS flow and the scratch assay. Our findings indicated that the increase in inlet flow rate in the mechanical assay led to a reduced experiment time and mechanical force on the cells, which could improve cell migration. Furthermore, the study on the effect of the device geometry showed that the increased channel width had an inhibitory effect on cell migration. The bi-functional chip offers an opportunity for the development of new models for a better understanding of cellular adhesion and migration in in vitro microenvironments.

## 1. Introduction

Microglia cells are innate resident immune cells of the central nervous system (CNS). Their wide range of functions, such as synaptic pruning and phagocytosis of apoptotic cells [1,2] in the CNS, always involve migrating under pathophysiological conditions [3]. They are the fastest-moving elements in the brain in their homeostatic condition [4]. Under pathological conditions, microglia cells rapidly become activated and migrate to sites of damage and inflammation. They take up pathological elements and tissue debris or secrete many soluble factors to repair the damaged tissue [5,6]. However, overactivation is deleterious in neurodegenerative diseases [7,8], brain cancers [9], and traumatic brain injury [2]. The microglial adhesive strength and behavior on the underlying surface play a central role in their migratory behavior, as cell adhesion to the surface enables microglia cells to crawl during their migration [10,11]. Therefore, a better understanding of microglia migration and adhesion can provide novel diagnostic, prognostic, and therapeutic strategies to control microglial activities in different brain states.

There are several techniques to characterize cell migration and adhesion quantitatively. Typical quantitative cell adhesion assays are the centrifugation assay [12,13,14] and the spinning assay [15,16,17]. In these assays, the fraction of detached cells was characterized by the loading force applied to the attached cells on the substrate [18]. Although their experiments are simple and usually available in laboratories, their performance is low throughout, with no real-time analysis probability [18,19,20].

For quantitative analysis of cell migration, the scratch assay is a common method [21,22,23,24,25]. This method creates a cell-free area (wound) by scratching over a confluent cell monolayer with a pipette tip. By introducing the cell-free gap to the cells, the cells start to migrate into the wound region. The migration activity of the cells is quantified by time-lapse imaging. This migration assay is convenient, easy to use, and enables us to monitor the cells regularly. However, scratching removes the surface coatings and damages the cells mechanically at the edge of the cell-free region (mechanical assay). The damaged cells at the edge of the cell-free region release intracellular contents, such as growth factors and cytokines, affecting cell migration [26,27]. To overcome these limitations, the barrier (stopper) wound-healing assay was utilized. In this assay, a barrier was kept on the substrate, and the cells were seeded around the barrier. After forming a confluent cell monolayer, the barrier was removed to create the cell-free gap. Nevertheless, the stopper could not mimic physical damage to the cells, such as trauma [28,29,30].

Microfluidic devices have been utilized to characterize cell adhesion and migration [19,31,32]. These devices with minimal sample volumes provide a more flexible design, real-time analysis, and precisely controllable cellular microenvironments [33,34]. These micro-devices, as cell adhesion assays, apply the flow within microchannels to expose the cells to mechanical fluid force (shear stress). Then, the fraction of adhered cells is characterized quantitatively. This method was used to study the cell adhesion on the substrates for human breast cancer cells (MCF10A and MCF7) [35], vascular and valvular endothelial cells [36], NIH/3T3 mouse fibroblast and bovine aortic endothelial cells [20], and fibroblast cells [37]. 

Furthermore, the microfluidics technique is capable of mimicking the wound-healing migration assay in a cost-effective and high-throughput manner with/without external stimuli [10,38,39]. For instance, Zhang et al. [40] proposed a microfluidic wound-healing assay that had a similar mechanism to the barrier wound-healing assay. It was fabricated with two layers of polydimethylsiloxane (PDMS). Once the cells were confluent, by peeling off the top PDMS layer with a circular pillar structure, circular cell-free regions were created. However, the device became an open chamber without the top PDMS layer, which could not quantify cell migration under the fluid flow (mechanical stimuli). Then, microfluidic wound-healing assays were multi-layered devices integrated with pneumatic actuators [41,42,43] and polymethyl methacrylate (PMMA) clamps [44]. The cell-free space was generated by deflecting/compressing the pneumatic and PDMS valves. Although these migration assays were automated, reproducible, and high-throughput, the different fabrication steps and control of their pressure and actuators made them complicated and time-consuming. In the most common method of microfluidic wounding, the cell-free area was created when the laminar trypsin and cell media flowed over the confluent cell monolayer in the microchannel. While the cells exposed to the trypsin solution were washed over the substrate, the cells in contact with the cell media remained attached [45,46,47,48,49,50,51,52,53,54]. This method is controllable in terms of the wound width and induces external stimuli over the cells in the laminar flow range. To drive the flow within the device, the device is integrated with a syringe pump that is not portable and is typically unavailable in biology laboratories. To remove the syringe pump, gravity is applied as a driving force to flow the trypsin within the device [55,56]. However, due to chemical (enzymatical) cell removal in this approach, the assay cannot generate wounds mechanically to mimic physical injuries (mechanical assay).

In the current study, we introduced a bi-functional microfluidic assay capable of quantifying the mechanobiological nature in terms of the adhesion and migration of microglial BV2 cells. As a wound-healing migration assay, this microfluidic device, with its simple microstructure and fabrication, can mimic any conditions between chemical and mechanical wound-healing migration assays. During mechanical wounding, the cell removal was characterized to quantify the cell adhesion under various shear stress ranges of PBS flow driven by the syringe pump. In chemical wound generation, laminar trypsin flowed within the device using gravity without any peripheral equipment (such as an external pump and microfluidic tubing). Cell migration was studied under chemical and mechanical wounding and different stress patterns, and then compared with the scratch assay. 

## 2. Materials and Methods

### 2.1. Device Design and Fabrication

**Figure 1:**
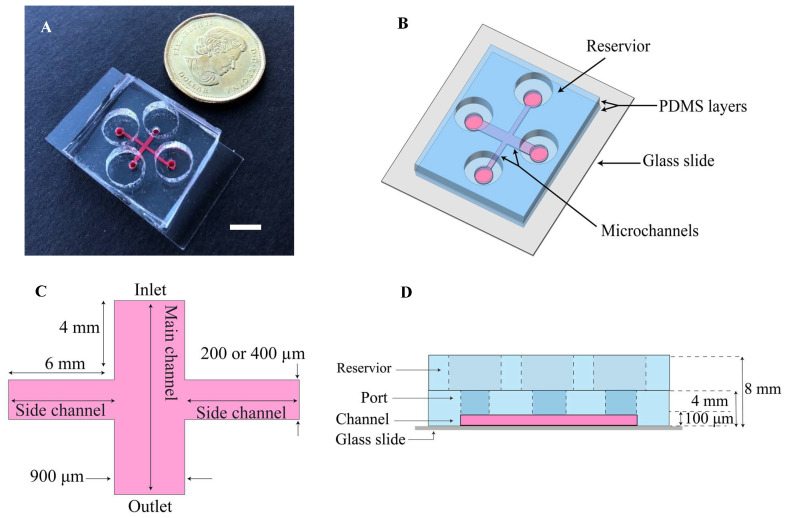
Schematic of the microfluidic assay and process of wound-healing in the device. (**A**) Photo of the microfluidic device with the microchannels filled with red dye (scale bar = 8 mm). (**B**) The layout of the device: (**C**) Top view and (**D**) side view show the device dimensions.

The device has intersecting channels, consisting of the main channel (width = 0.9 mm, length = 8.2 or 8.4 mm) and side channels (width = 200 or 400 µm, length = 6 mm) (Figure 1A–D). The devices made of PDMS were fabricated using soft lithography with replica molding. The master mold was on a silicon wafer (WRS material, Alpha Nanotech Inc., Virgin, USA) using a negative photoresist pattern (SU8-2075, MicroChem, Round Rock, TX, USA). To make a mold with 100 µm thickness, the silicon wafer was spin-coated with a layer of SU8-2075, pre-baked, exposed to UV light, and post-baked. The master was made when the unexposed photoresist on the mold was washed with the SU-8 developer. The PDMS, a mixture of the elastomer base (Sylgard 184, Dow Corning, Midland, MI, USA) and the elastomer curing agent (Sylgard 184, Dow Corning, Midland, MI, USA) at a ratio of 10:1, was poured over the master and incubated at 70 °C for 2 h. After peeling off the cured PDMS over the mold, the terminals of the channels were punched to make ports with a diameter of 1.5 mm. The PDMS (4 mm in height) was irreversibly bonded onto the microscope glass coverslip using oxygen plasma treatment for 90 s. Then, again, the upside of the PDMS layer was treated to bond to another PDMS (top layer, 4 mm in height), which includes four media reservoirs punched with a diameter of 8 mm (Figure 1D). This paper used simple optical methods for detection, such as optical microscopy, inverted microscopy, and an area-counting method using images.

### 2.2. Cell Culture

The murine microglial BV2 cells (~5 × 10^5^ cells/mL concentration, passage 14, gifted from Dr. Carol Colton, Duke University) were cultured in Dulbecco’s Modified Eagle’s Medium (DMEM; Wisent Technologies, Saint-Bruno, QC, Canada) supplemented with 10% fetal bovine serum (FBS, Invitrogen, TX, USA), 1% Penicillin-streptomycin (Wisent Technologies, Saint-Bruno, QC, Canada) until the cells reached confluence [57].

### 2.3. Cell Seeding

The cells were harvested with 0.05% trypsin (Wisent Technologies, Saint-Bruno, QC, Canada). After centrifuging the cells at 1100 rpm for 8 min, the supernatant was removed, and the cells were resuspended by adding 1 mL of fresh cell media. Before the cell seeding into the channels, the device was sterilized with UV light for 30 min. The cells in the device were seeded as follows: the cells (~1 × 10^7^ cells/mL) were injected into the device from the inlet port using a syringe (1 mL, Becton, Dickinson and Company, Franklin Lakes, NJ, USA) connected to a needle (16G1/2, Becton, Dickinson and Company, NJ, USA). Then, three pipette tips (20 µL, FroggaBio, Vaughan, ON, Canada) were inserted into the other three ports (the outlet and side channel terminals) to trap the cells in the device with a higher cell concentration. The syringe was gently removed from the inlet. Next, the device was left in the incubator. After 30 min, when the cells were attached to the substrate, the pipette tips were gently removed, and the reservoirs were filled with 150 µL of fresh cell medium [58]. 

### 2.4. Experimental Setup for Wound Edge Formation

**Figure 2:**
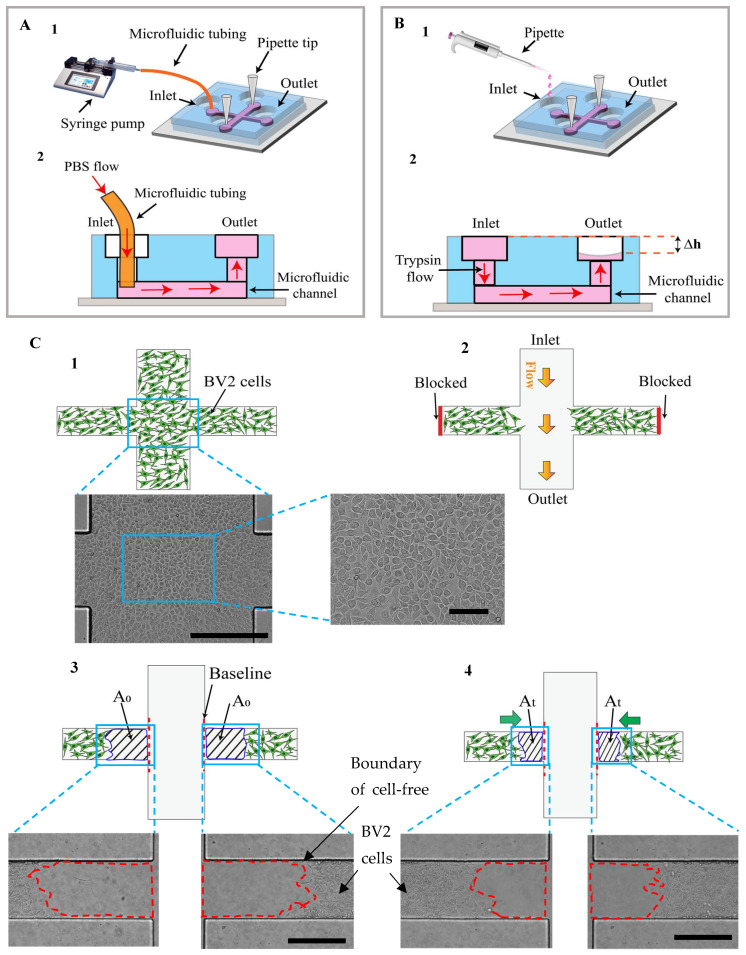
There were two experimental setups for the cell-free creation using the microfluidic device: (**A**) Active-PBS method and (**B**) Passive-Trypsin method. (**C**) The cell-free area was generated as follows: (**C1**) microglial BV2 cells reach confluence within the device (scale bar = 400 µm). (**C2**) While the side channels were blocked, trypsin or PBS flow was driven from the inlet to the outlet to wash the cells in the main channel. (**C3**) After unblocking the side channels, the initial cell-free area was introduced (the wound edge to the baseline, A_0_). (**C4**) The cells began to migrate to the cell-free area. The cell migration area was obtained by subtracting A_0_ from the cell-free area at the desired time (A_t_) (scale bar= 400 µm).

The cell-free area was created with two general approaches. In the first approach, the laminar flow of PBS was pumped into the main channel using a syringe pump (active pumping) in two chips with side channel widths of 200 and 400 µm (Figure 2(A1,2), called the Active-PBS-200 and Active-PBS-400 methods, respectively). In another approach, the laminar trypsin flow was driven within the device to generate the cell-free space using gravity (passive pumping) in two chips with side channel widths of 200 and 400 µm (Figure 2(B1,2) called the Passive-Trypsin-200 and Passive-Trypsin-400 methods, respectively).

The cell-free area was created in the chip when a confluent cell-monolayer was formed (Figure 2(C1)). In the Active-PBS method (Figure 2(A1,2)), the pipette tips (the pipette tips were blocked by PDMS) were inserted into the side channel ports to block the terminals of two side channels (Figure 2(A1,C2)). Then, the cell media in the reservoirs were removed. A 10 mL syringe containing 0.01X PBS was mounted onto the syringe pump (Legato 111, KD Scientific, Holliston, MA, USA). The syringe was connected to the inlet by Fluorinated Ethylene Propylene (FEP) tubing (inner diameter, 0.5 mm; outer diameter, 1.6 mm, Figure 2(A1,2)). Then, PBS was pumped into the device with the inlet Re (Reynold number) of 25, 50, and 100 for 5, 3, and 2.2 min, respectively (Figure 2(C2)). When all the cells in the main channel were detached (Figure 2(C3)), the tubing and the pipette tips were gently removed. Then, PBS in the channels was washed out by adding the media to the reservoirs, and the device was left in the incubator (37 °C and 5% CO_2_). 

In the Passive-Trypsin method, the cell-free area was generated as follows: after blocking the terminals of the side channels (ports) once the cells reached confluence (Figure 2(C1)), the cell media of the reservoirs were aspirated. Then, the trypsin flowed from the inlet to the outlet by adding 200 µL of trypsin to the inlet and 50 µL of trypsin to the outlet (to overcome the surface tension resistance in the outlet), respectively (Figure 2(B1,2, C2). The device was incubated (37 °C and 5% CO_2_) for 9 min. Then, when all the cells in the main channel were washed (Figure 2(C3)), the trypsin was aspirated from the reservoirs. 150 µL of cell media was added to each reservoir to wash and neutralize the trypsin in the channels. Finally, the device was kept in the incubator (37 °C and 5% CO_2_). 

### 2.5. Simulation of Fluid Flow Behavior in the Device

The shear stress acting on the cells in the microfluidic device was computed using the ″Laminar Flow″ 3D module of COMOSL Multiphysics 5.5 for the Active-PBS method. The steady-state Navier–Stokes equations were solved to determine the flow field. The fluid flow within the device was assumed to be laminar, viscous, Newtonian, and incompressible. The dynamic viscosity (μ, 0.00845 Pa.s) and density (ρ, 996 kg/m^3^) were assumed to be the same as deionized water (DIW) at 27 °C. The channel walls and outlet boundary conditions were defined as no-slip and atmospheric pressure with normal flow, respectively [59]. In the Active-PBS method, the inlet boundary condition was kept at inflow velocities for Re = 25, 50, and 100. The average inlet velocity (v) for Passive-Trypsin was measured by a video taken with a camera (3 Megapixel CMOS). The video was taken from the side view of the inlet reservoir, which showed the discharging of the liquid within the device using gravity (Supplementary Movie). By measuring the liquid level every 20 s, the average inlet velocity was calculated by (VOL_t−20_ − VOL_t_)/A_i_, where VOL was the liquid volume of the inlet reservoir at each time step (t) of 20 s (t started from 20 s) and A_i_ was the inlet area of the main channel. The Re corresponding inlet was defined as follows,

(1) $$Re=\frac{\rho vD}{\mu }$$

where D was the hydraulic diameter of the main channel.

The shear stress (τ) in the microfluidic assay was measured as follows [57,60],

(2) $$\tau =\frac{6µ\mathrm{Uave}}{h}\left(1-\frac{2y}{h}\right)$$

where U_ave_ was the average velocity, µ was the fluidic viscosity, h was the height, and y was the distance from the bottom plate. The shear stress and velocity streamlines were studied at a height of 3 µm, considering the height of microglial cells [61]. The idea of this model is to predict the shear stress acting on the cell, leading to cell removal. To measure the average shear stress on the residual cells of the side channel in wounding, the simulated shear stress along 10 locations, from the edge cells to the side channel terminal, was averaged.

### 2.6. Scratch Assay

Microglia BV2 cells (5 × 10^5^ cells/well) were seeded onto the sterilized microscope glass coverslips (22 × 22 mm, Fisher Scientific, Hanover Park, IL, USA) left in a 6-well plate. The scratch was made over the confluent cell monolayer by a sterile 1 mL pipette tip. Then, the cells were gently washed with PBS 0.01X. After adding 2 mL of cell media to each well, the well plate was incubated at 37 °C and 5% CO_2_ [62,63]. 

### 2.7. Data and Statistical Analysis

The images were taken using an inverted fluorescence microscope (4× and 10× magnification objective, AMG EVOS FL). After creating the wound, the images were captured at 0, 6, 12, 18, and 24 h time points. Image J 6.0 software was used to measure the cell-free area.

The cell-free area in the side channel (A) at each time point was defined from the wound edge to the baseline (Figure 2(C3,4)). The average cell migration distance (δ_t_) at each time point (t) was expressed as follows:

δ_t_ = (A_0_ − A_t_)/W (3)

where A_0_ and A_t_ were the cell-free area in the side channel at the initial and the desired time (t), respectively (Figure 2(C3,4)), and W was the side channel width. The cell migration rate (µ_r_) at each time point was obtained by the following expression [53,64]:

µ_r_ = δ_t_/t (4)

The cell migration distance in the scratch assay was calculated as follows:

δ_t_ = (S_0_ − S_t_)/2H (5)

where S_0_ and S_t_ were the cell-free areas between two wound edges in the image at the initial and desired time (t), respectively, and H was the height of the analyzed image.

The quantified data were expressed as the mean ± standard deviation (SD). The cell-free area in the side channel measured at each time point was the average of the cell-free area in the left and right side channels. Each experiment was repeated three times. The statistical analysis, ANOVA, was performed using GraphPad PRISM 5. *p* < 0.05 (*: *p* < 0.05, **: *p* < 0.01, ***: *p* < 0.001, and ****: *p* < 0.0001) was considered statistically significant.

## 3. Results and Discussion

### 3.1. Conceptual Design of the Microfluidic Adhesion and Migration Assay

A microfluidic device was developed to study cell adhesion and migration mechanically and chemically in assays. In the mechanical assay (Active-PBS method), the mechanical fluid force was created through fluid shear stress on the cell by varying the flow velocity; when the fluid load exceeded cell adhesive strength, the cells were detached from the substrate. In the chemical assay (Passive-Trypsin method), trypsin was used to deactivate the cell adhesion and detach the cells from the substrate. 

The microfluidic adhesion assay functioned as a mechanical wounding assay during cell-free generation (Active-PBS method). Cell adhesion was measured by quantifying cell detachment under a wide range of shear stress. 

The microfluidic wound-healing migration assay performed mechanical and chemical wounding assays to generate the cell-free area (the Passive-Trypsin method and the Active-PBS method). The cross-channel geometry enabled us to create the cell-free area in the main channel over the confluent cell monolayer, while the cells in the side channels were kept attached (Figure 2(C2,3)). In order to achieve this aim, during wounding, the pressure of the side channels compared to the main channel was increased by blocking the side channel terminal and increasing the main channel width (900 µm) to be higher than the side channels (200 or 400 µm). The pressure of the side channel was higher than the main channel, leading to the trypsin or PBS flowing only in the main channel and reducing the fluid flow to the side channels. Therefore, the cells in the main channel were removed chemically with trypsin and mechanically with PBS, while the majority of the cells in the side channels were kept attached. Furthermore, in the Passive-Trypsin method, due to the cross-channel geometry of the device, when the trypsin flowed from the inlet to the outlet, the residual cell media in the side channels was trapped. It helped to reduce the cell removal in the side channels when the trapped cell media neutralized the trypsin effect in the side channel. 

In the Passive-Trypsin method, gravity was used to drive the trypsin solution within the device for cell-free creation. Therefore, despite previous methods, for the wounding, peripheral equipment was not required (such as a pneumatic pump [41,42,43], a syringe pump, or microfluidic tubing [40,41,42,43,44,45,46,47,48,49,50]), making the cell migration quantification cost-effective. Furthermore, in the Active-PBS method, the cells were detached in the main channel through the fluid shear due to PBS flow. It allowed for the investigation of cell adhesion dynamics during the wounding process and the effect of mechanical stress on cell migration.

### 3.2. Mechanism of Cell-Free Area Generation

**Figure 3:**
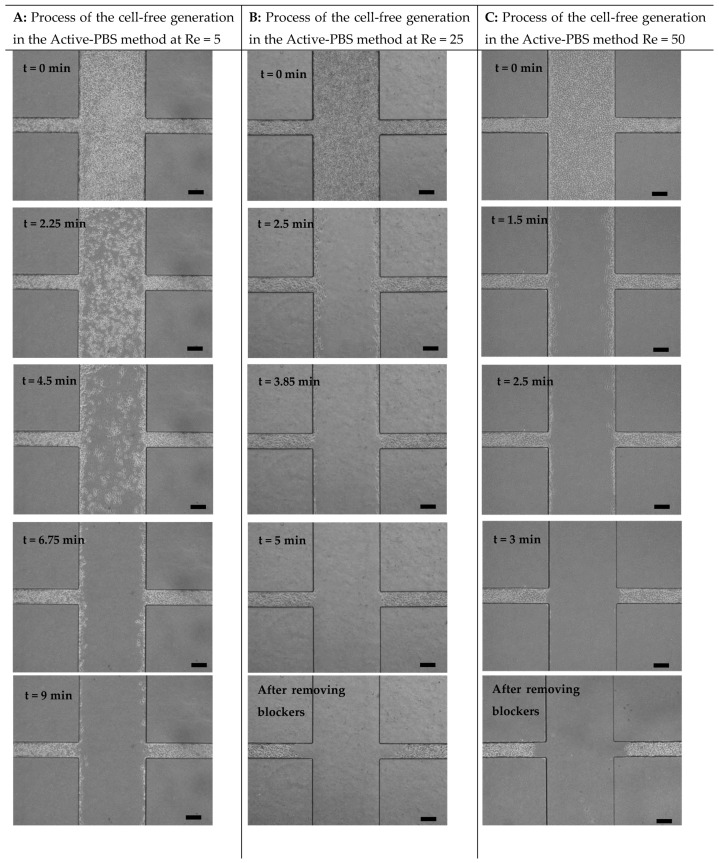

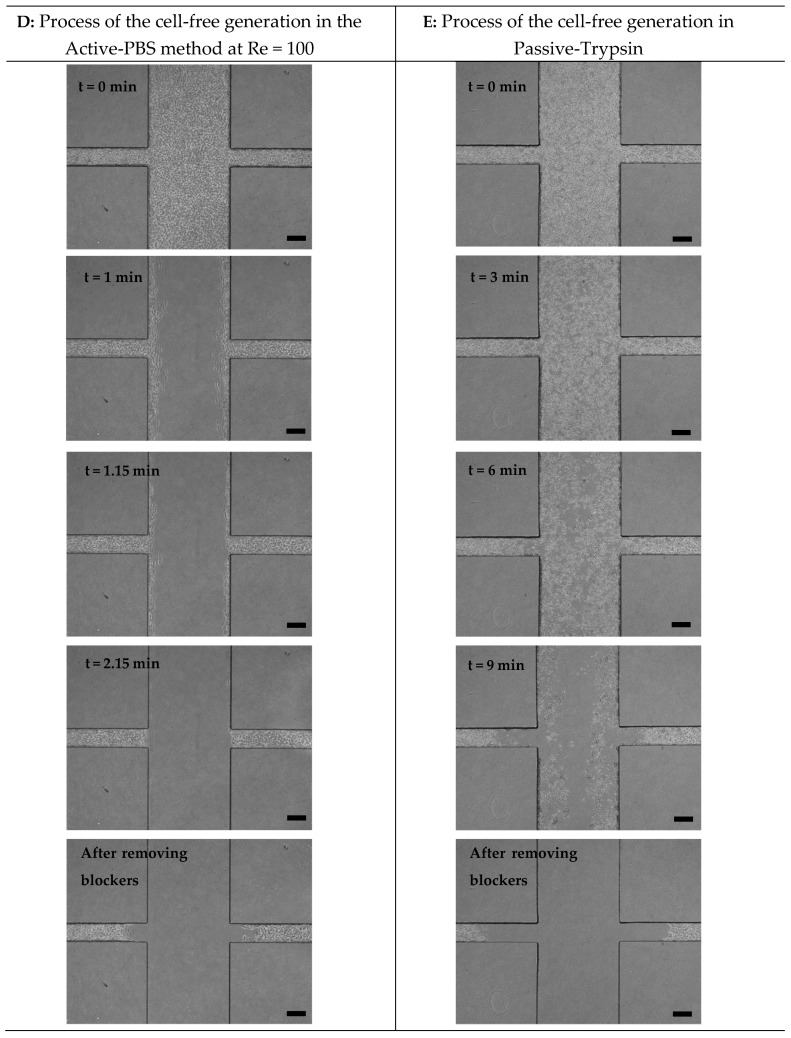
Process of the cell-free generation for the Active-PBS method at (**A**) Re = 5, (**B**) Re = 25, (**C**) Re = 50, and (**D**) Re = 100 and for (**E**) the Passive-Trypsin method. Scale bar = 200 µm.

In order to create the cell-free area, the experimental parameters, such as the rates of PBS flow, the volume of trypsin used in the inlet and outlet, and the flow duration, were optimized. Figure 3 shows the process of cell-free creation in the Active-PBS-200 methods and the Passive-Trypsin-200 method through time-sequence microscope images. In the Active-PBS method at the inlet corresponding to Re of 5, the shear stress produced by PBS flow using the syringe pump was not enough to detach whole cells in the main channel, even after 9 min (Figure 3A). By increasing the flow at the inlet Re of 25, 50, and 100, the cells in the main channel were removed after 5, 3, and 2.2 min, respectively (Figure 3B–D). In the Passive-Trypsin method, to drive the trypsin flow from the inlet to the outlet by gravity force, a different hydrostatic pressure was created by adding 200 µL trypsin to the inlet and 50 µL trypsin to the outlet (Figure 2(B1,2)). After 9 min, the cells in the main channel were washed with cell medium (Figure 3E). Increasing the volume to more than 200 µL trypsin in the inlet could not form a clear wound edge in the side channels. It means that a few cells were not detached from the side channel walls, while the cells in the center of the side channel were removed. Furthermore, without adding 50 µL trypsin to the outlet, the surface tension resistance in the outlet did not allow flowing trypsin in the main channel. 

### 3.3. Quantitative Analysis of Cell Adhesion

In order to investigate the adhesion of microglia BV2 cells quantitively during the cell-free generation, the duration and fraction of the cell removal under a wide range of shear stress were quantified using the Active-PBS method. 

**Figure 4:**
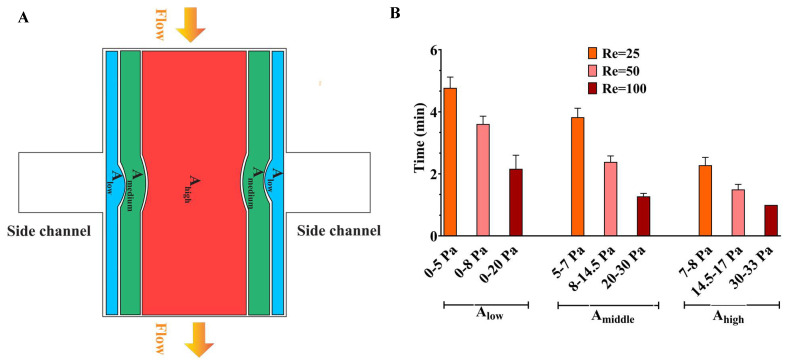
(**A**) The areas in the main channel were categorized based on the sequence of cell removal during wounding. Initially, the cells in A_high_ (1,440,000 µm^2^ with ~1870 cells) were removed, and next A_medium_ (240,000 µm^2^ with ~310 cells), and finally A_low_ (120,000 µm^2^ with ~155 cells). (**B**) The duration for cell removal under different shear stress ranges at Re = 25, 50, and 100.

Once the cell removal pattern was studied, it was noticed that the cell removal area in the main channel occurred in a common sequence at various flow rates when initially the cells in the area under high shear stress, A_high_, were detached, then the area under medium shear stress, A_medium_, and finally the area under low shear stress, A_low_ (Figure 4A). The areas of A_high_, A_medium_, and A_low_ were approximately 1,440,000 µm^2^ (~1870 cells), 240,000 µm^2^ (~310 cells), and 120,000 µm^2^ (~155 cells), respectively. The shear stress range selected for defining high, medium, and low stress areas is shown in Table 1 for various Re. Table 2 demonstrates the zones of the main channel corresponding to high, medium, and low shear stress ranges in different Re.

Figure 4B indicates that ~80% of cells (~1870 cells in A_high_) in exposure to 30–34 Pa (Re = 100), 14.5–17 Pa (Re = 50), and 7–8 Pa (Re = 25) were removed after 1, 1.49, and 2.27 min, sequentially. ~13% of cells (~310 cells in A_medium_) under shear stresses of 20–30 Pa (Re = 100), 8–14.5 Pa (Re = 50), and 5–7 Pa (Re = 25) were removed after 1.27, 2.38, and 3.82 min, respectively. The removal times for ~7% of cells (~155 cells in A_low_) were 2.71, 3.6, and 4.77 min, subjected to shear stresses of 0–20 Pa (Re = 100), 0–8 Pa (Re = 50), and 0–5 Pa (Re = 25), respectively. The results show that the time of cell removal is strongly related to the local shear stress when higher shear stress removes the cells in less time. Our findings indicate that the detachment force and duration required for microglia BV2 cells in the control condition can be used as a quantitative measure to be compared with pathological conditions. 

### 3.4. Investigation of Fluid Flow Behavior in the Microfluidic Device

**Figure 5:**
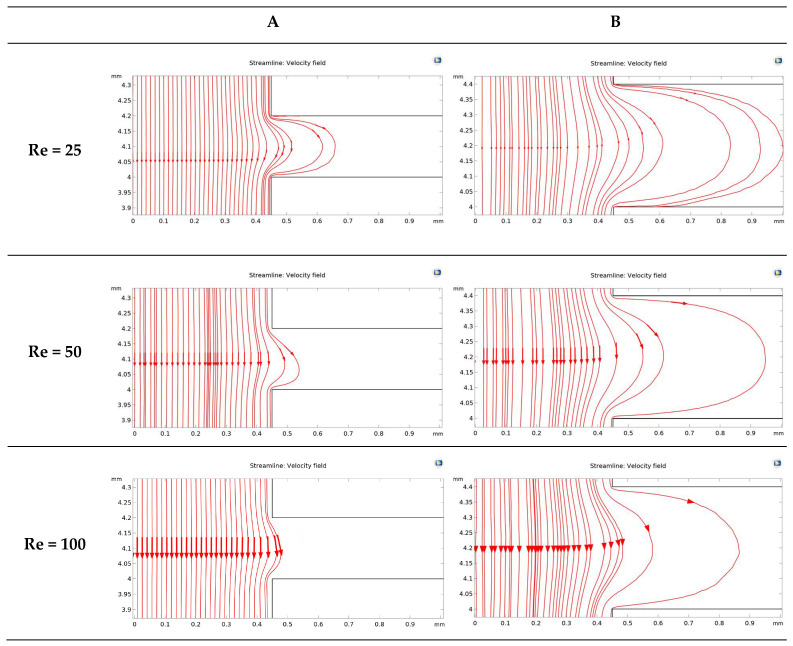
Velocity streamline for the devices with a side channel width of (**A**) 200 µm and (**B**) 400 µm at Re = 25, 50, and 100.

The fluid flow behaviors during cell-free generation can affect cell mobility in the side channel after wounding. This section studies the fluid flow dynamics in the chips with two side channel widths (200 and 400 µm) at Re = 25, 50, and 100 (the Active-PBS-200 and 400 methods). At lower Re, the fluid streamed deeper into the side channel for both widths of the channel, 200 and 400 µm (Figure 5A,B). At low Re, the flow was more laminar, leading to moving fluid higher to the side channel. Furthermore, the increase in Re at the inlet created a higher pressure difference between the inlet and outlet; therefore, with higher Re, there was more tendency for the fluid to keep flowing in the main channel. 

In the following sections, the effect of fluid flow dynamics and the side channel width on cell migration will be experimentally studied. 

### 3.5. Quantification of Cell Migration in the Process of Cell-Free Area Generation Chemically and Mechanically

**Figure 6:**
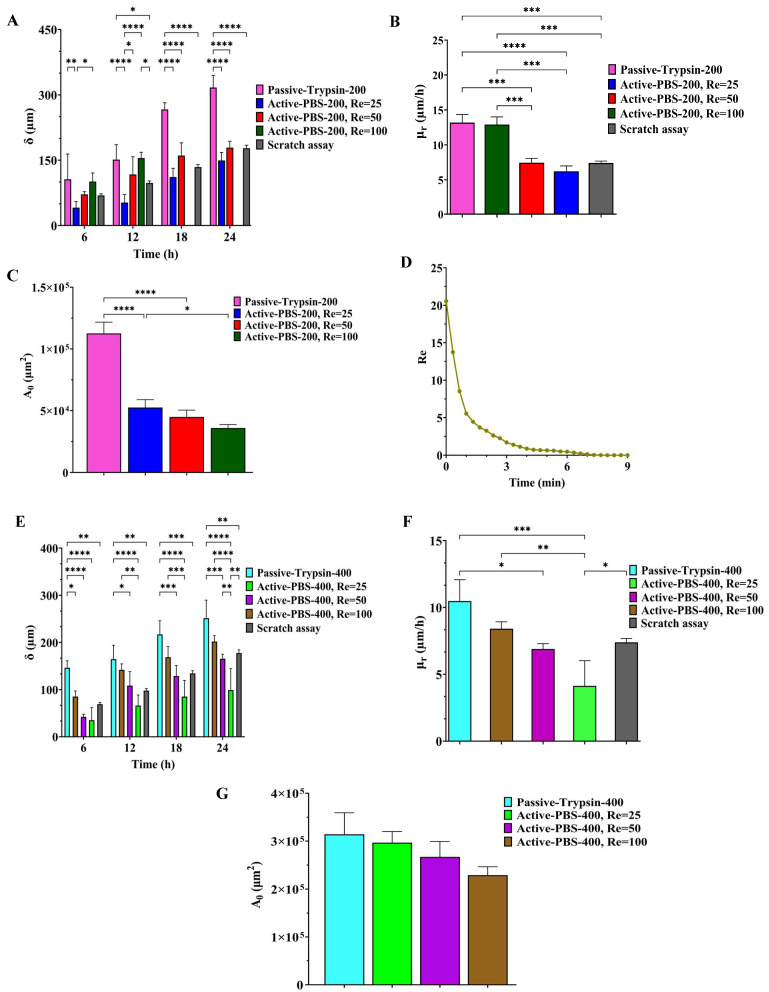
Quantification of (**A**) cell migration distance (δ), (**B**) cell migration rate (µr), and (**C**) the initial wound area in the side channel after wounding for the Passive-Trypsin-200 method and the Active-PBS-200 method at Re = 25, 50, and 100. (**D**) Re corresponding to the inlet of the main channel in the Passive-Trypsin-200 method during wounding. Quantification of (**E**) cell migration distance (δ), (**F**) cell migration rate (µr), and (**G**) the initial wound area in the side channel after wounding for the Passive-Trypsin-200 method and the Active-PBS-400 method at Re = 25, 50, and 100. Data represent the mean ± SD of three independent experiments (*n* = 3). ANOVA was used for statistical analysis, *: *p* < 0.05, **: *p* < 0.01, ***: *p* < 0.001, and ****: *p* < 0.0001.

In order to characterize the effect of both mechanical and chemical methods of creating the cell-free area on cell migration, microglial BV2 migration was quantified using the Passive-Trypsin-200 method and Active-PBS-200 method at Re = 25, 50, and 100 (Figure 6A,B).

**Figure 7:**
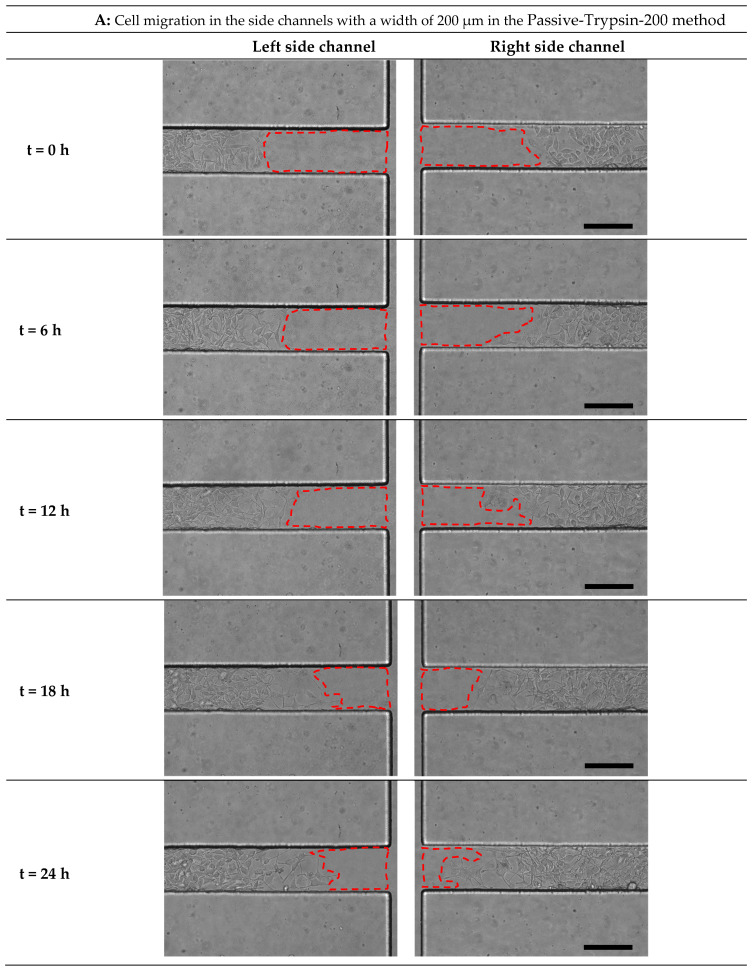

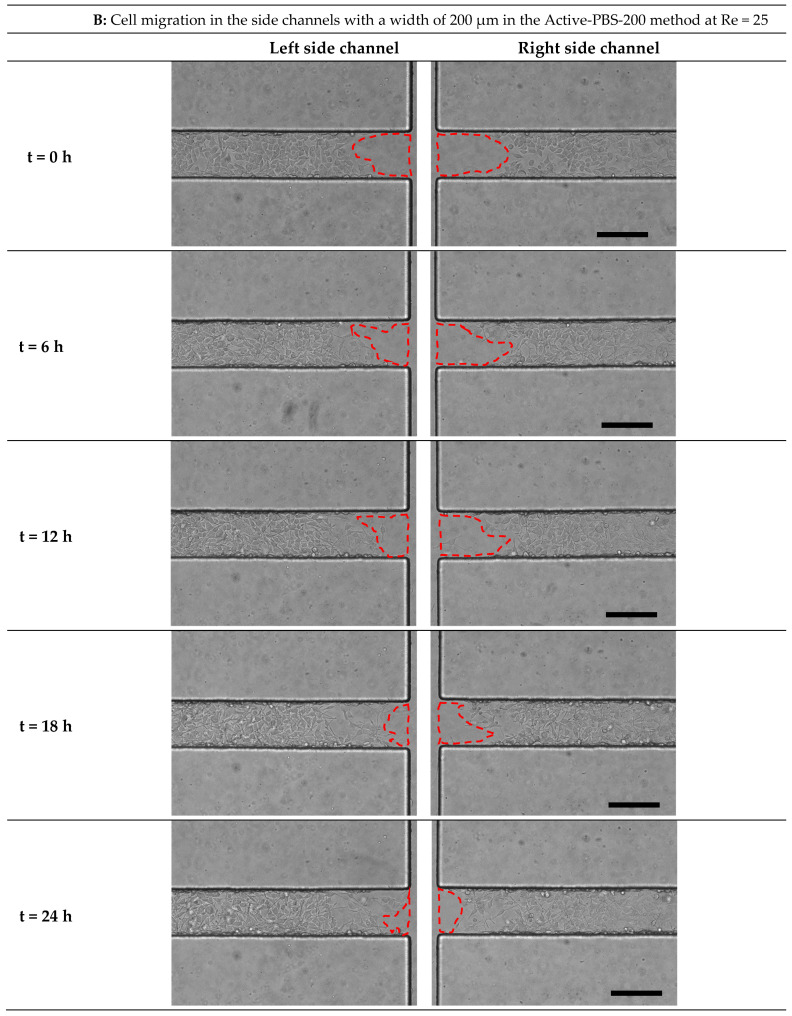

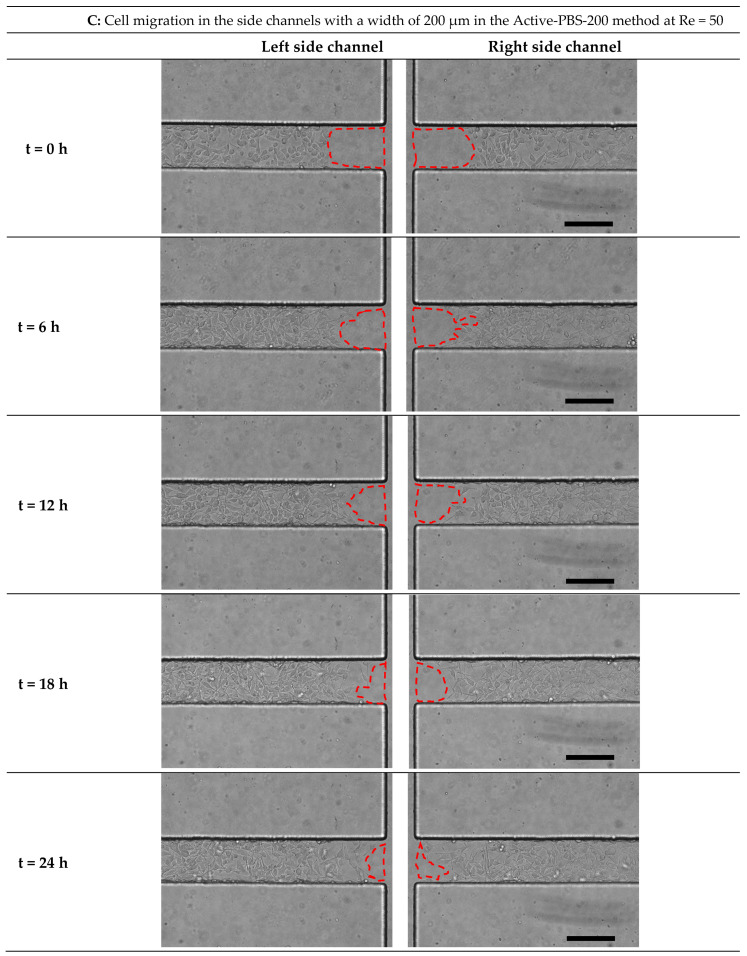

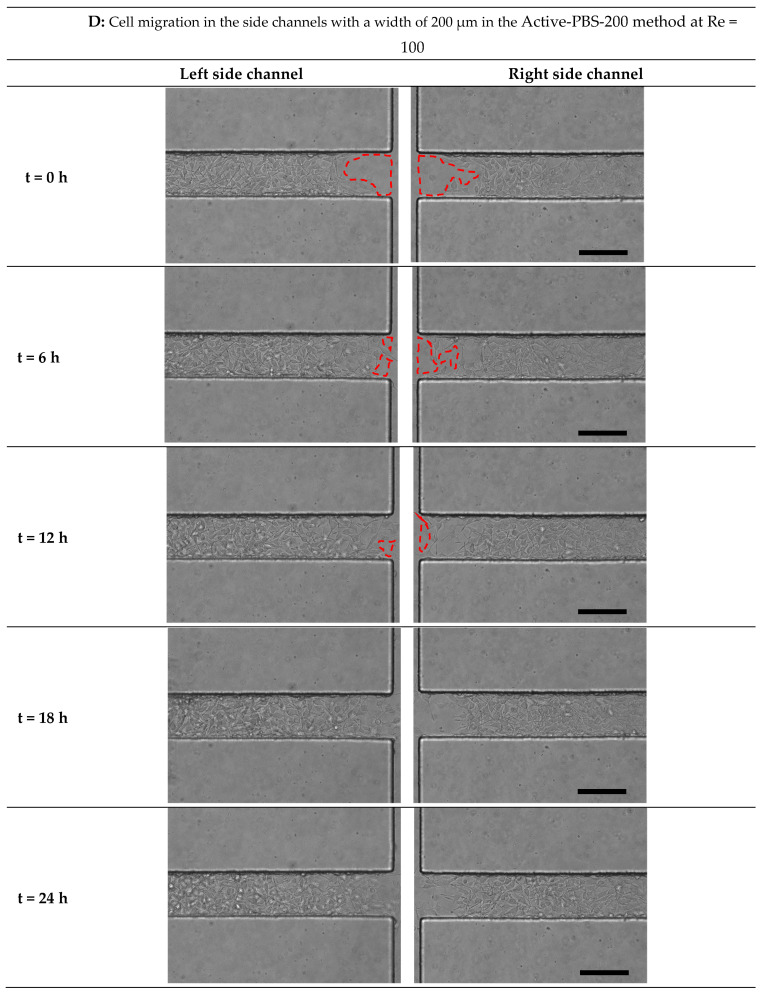
Time-lapse images from cell migration in the side channels with a width of 200 µm in (**A**) the Passive-Trypsin-200 method and the Active-PBS-200 method at (**B**) Re = 25, (**C**) Re = 50, and (**D**) Re = 100 at 0, 6, 12, 18, and 24 h after the cell-free generation. Scale bar = 200 µm.

**Figure 8:**
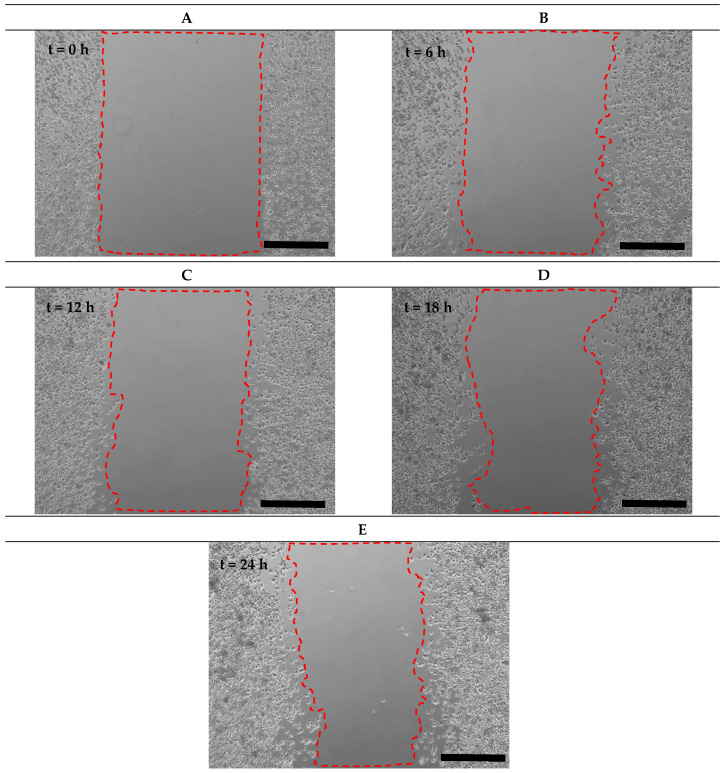
Time-lapse images from cell migration in the scratch assay at (**A**) 0 h, (**B**) 6 h, (**C**) 12 h, (**D**) 18 h, and (**E**) 24 h. Scale bar = 500 µm.

After creating the cell-free zone in the main channel, microglial BV2 migration to the cell-free area was imaged in the microfluidic migration assay and the scratch assay at 0 h, 6 h, 12 h, 18 h, and 24 h (Figure 7A–D and Figure 8). 

Cell migration was quantified from the edge cells of the cell-free area in the side channel to the baseline (Figure 2(C3,4)). If the cells passed the baseline, measuring cell migration would be difficult. For Active-PBS-200 method at Re=100, cell migration was measured until 12 h after the wounding. Because at 12 h, the cells reached the baseline, and at 18h, the cells passed the baseline (Figure 7D). 

In the Active-PBS-200 methods, increased inlet Re for cell removal enhanced cell migration. Cell migration for the Active-PBS-200 method at Re = 100 was 154.96 ± 13.23 µm after 12 h, higher than Re = 50 (117.29 ± 40.59 µm) and Re = 25 (52.76 ± 18.66 µm, *p* < 0.0001) (Figure 6A). Similarly, after 24 h, the Active-PBS-200 method at Re = 50 had a greater cell migration distance than Re = 25 (149.33 ± 18.70 vs. 178.68 ± 14.83 µm, Figure 6A), whereas the Passive-Trypsin-200 method with 316.91 ± 27.47 µm migration had the significantly highest migration than other cases (*p* < 0.0001) (Figure 6A).

By comparing the cell migration distance in the microfluidic migration assay with the scratch assay (Figure 6A), it was found that, at 24 h, the cell migration distance in the scratch assay (177.54 ± 6.75 µm) was almost equal to the Active-PBS-200 method at Re = 50 (178.68 ± 14.83 µm). Furthermore, the cell migration distance in the scratch assay was significantly less than the Passive-Trypsin-200 method (316.91 ± 27.47 µm, *p* < 0.0001) and greater than the Active-PBS-200 method at Re = 25 (149.33 ± 18.70 µm).

Figure 6B shows the migration rate of microglial BV2 cells after 24 h, except for the Active-PBS-200 method at Re = 100, which was quantified after 12 h of migration. The migration rate for the Passive-Trypsin-200 method was the highest (13.20 ± 1.14 µm h^−1^) compared to the Active-PBS-200 method at Re = 100 (12.91 ± 1.1 µm h^−1^), Re = 25 (7.44 ± 0.61 µm h^−1^, *p* < 0.001), Re = 25 (6.22 ± 0.77 µm h^−1^, *p* < 0.0001), and the scratch assay (7.39 ± 0.28 µm h^−1^, *p* < 0.001).

By reducing Re in the Active-PBS-200 method, the flow became more laminar and entered deeper into the side channel (Figure 5A), which caused the removal of more of the cell area in the side channel (52,563 µm^2^, 44,923 µm^2^, and 35,840 µm^2^ for the Active-PBS-200 method at Re = 25, 50, and 100, respectively, Figure 6C). It means that the cells in the side channel with lower Re were exposed to a higher mechanical shear force during cell removal, inhibiting cell migration. Also, once Re increased, the wounding time was reduced, leading to a reduced exposure time of the cells to mechanical force.

It was observed that the Passive-Trypsin-200 method had the highest cell migration. Because, after almost 3 min of wounding with trypsin, the trypsin flow in the main channel was a creeping flow with Re < 1 (Figure 6D). It shows that the cell removal in the Passive-Trypsin-200 method was mainly a chemical (enzymatical) interaction, and the mechanical force on the cells in the side channel was less significant. 

The migration results showed that the microfluidic chemical migration assay (Passive-Trypsin-200 method) enhances cell migration compared to the microfluidic mechanical migration assay (Active-PBS-200 method) and the scratch assay. The migration assay under high Re is very similar to the chemical migration assay due to less mechanical force on the cells in the side channels and resulting in less cell damage, while the migration assay under low Re is similar to the scratch assay due to the high mechanical force and damage to the cells. This shows that the chemical migration assay (chemical wounding) results in less mechanical damage and shock to the cells as the interaction is mainly chemical. However, in the scratch assay, there is mechanical interaction for cell removal (mechanical wounding); hence, mechanical damage would be higher. The microfluidic migration assay presents the possibility and variety of shear force on cells through flow velocity (Re) in creating chemical and mechanical woundings. 

### 3.6. Effect of Fluid Loading through the Geometry Variation of the Side Channel on Cell Migration

**Figure 9:**
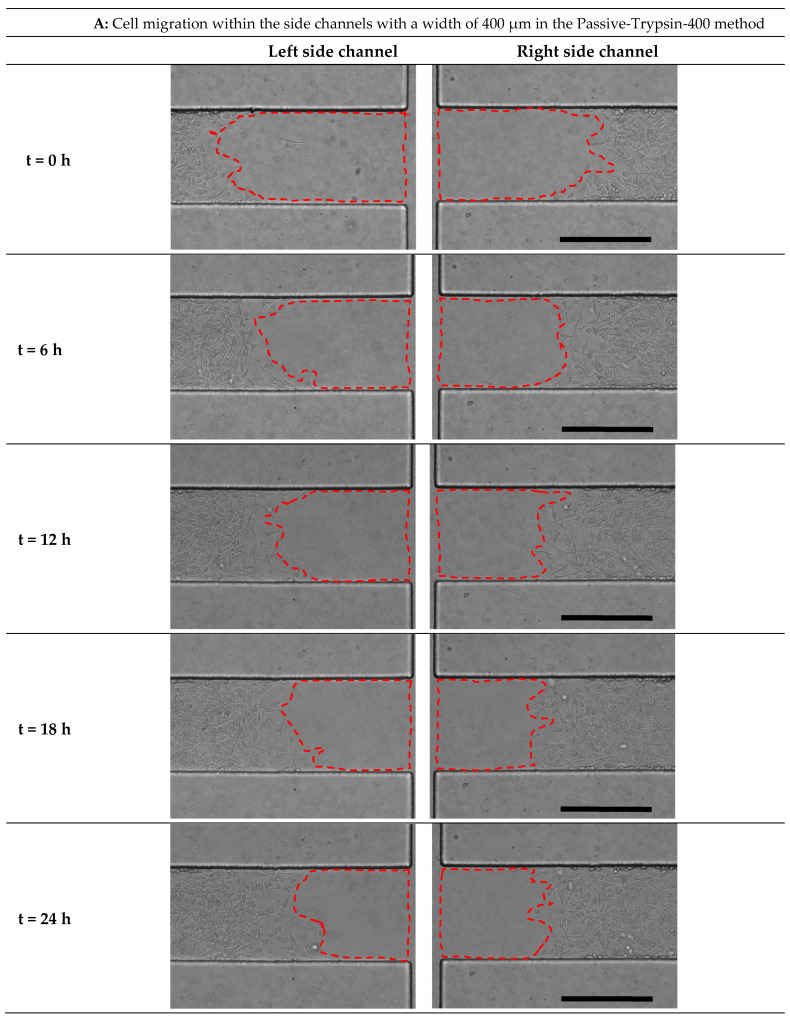

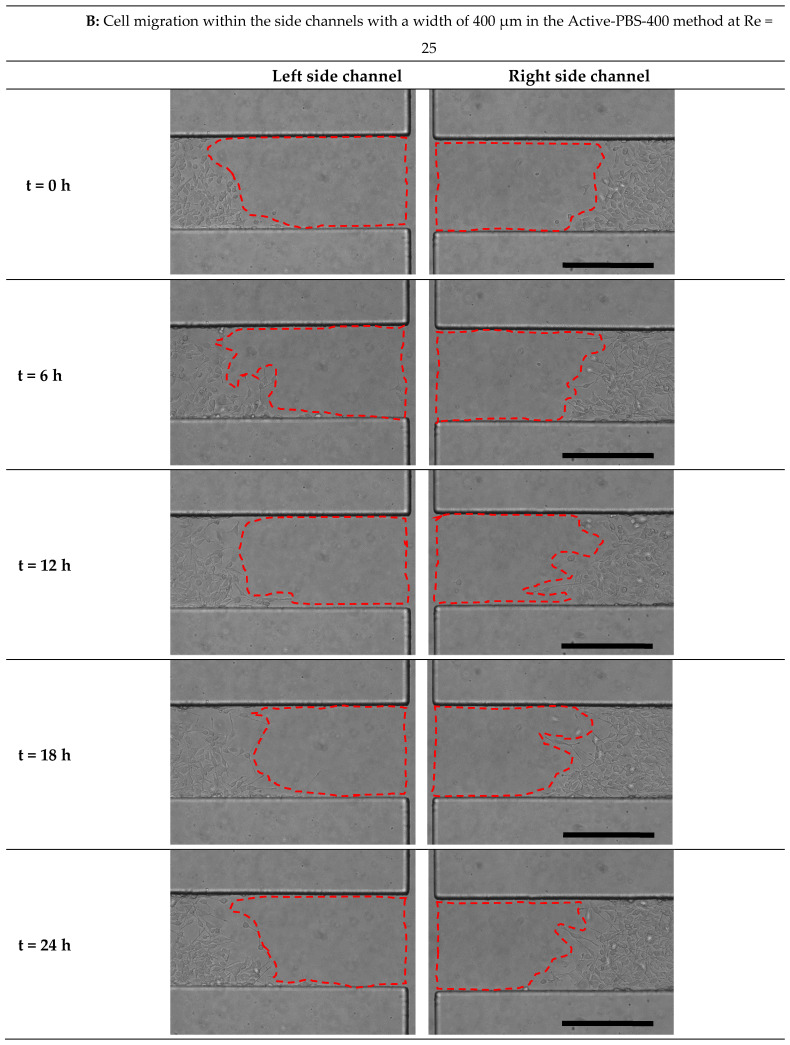

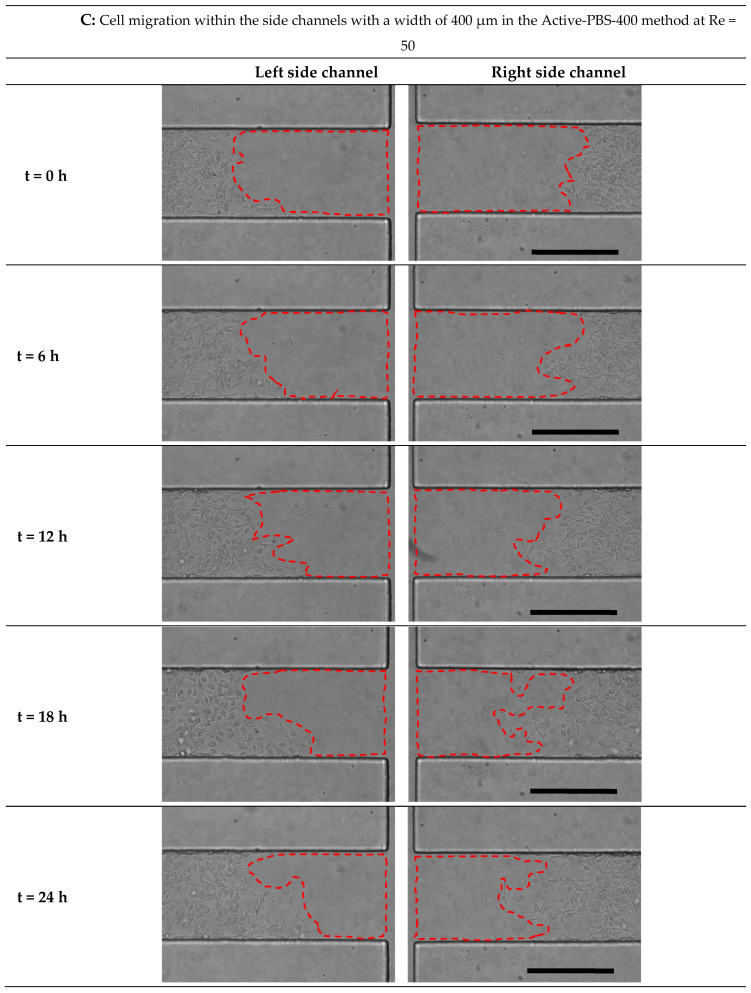

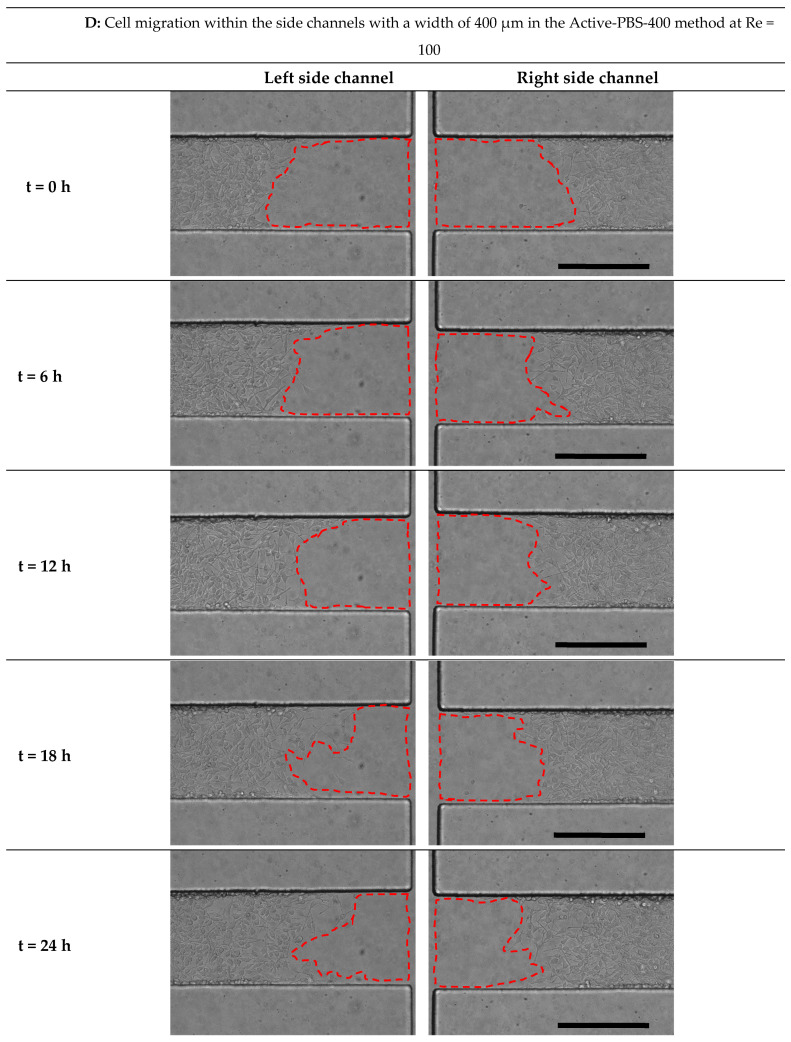
Time-lapse images from cell migration within the side channels with a width of 400 µm in (**A**) the Passive-Trypsin-400 method. The Active-PBS-400 method at (**B**) Re = 25, (**C**) Re = 50, and (**D**) Re = 100 at 0, 6, 12, 18, and 24 h after cell-free generation. Scale bar = 400 µm.

In order to create different stress patterns on cell migration, the side channel width was increased from 200 to 400 µm. Figure 9A–D illustrates cell migration in the chip, with a side channel width of 400 µm for the Passive-Trypsin-400 method and the Active-PBS-400 method at Re = 25, 50, and 100 at 0, 6 h, 12 h, 18 h, and 24 h after wounding. 

The cell migration trend in the device with a side channel width of 400 µm was the same as 200 µm when the Passive-Trypsin-400 method induced the highest migration, and increasing Re in the Active-PBS-400 method also enhanced cell migration (Figure 6E). 

After 24 h of migration, there was a significant cell migration distance for the Passive-Trypsin-400 method (251.55 ± 38.25 µm) compared to the Active-PBS-400 method at Re = 25 (114.26 ± 53.38 µm, *p* < 0.0001), Re = 50 (165.39 ± 9.6 µm, *p* < 0.01), and Re = 100 (201.903 ± 12.36 µm, Figure 6E). Cell migration in the scratch assay after 24 h reached 177.54 ± 6.75 µm, higher than the Active-PBS-400 method at Re = 25 (*p* < 0.5) and Re = 50, and less than the Passive-Trypsin-400 method (*p* < 0.01) and the Active-PBS-400 method at Re = 100 (Figure 6E).

Figure 6F demonstrates the migration rate of microglial BV2 cells in the device with a side channel width of 400 µm and the scratch assay at 24 h. The migration rate of microglial BV2 cells for the Passive-Trypsin-400 method (10.48 ± 1.59 µm h^−1^) was a significant difference from the Active-PBS-400 method at Re = 25 (4.13 ± 1.88 µm h^−1^, *p* < 0.01) and 50 (6.89 ± 0.4 µm h^−1^, *p* < 0.5) at 24 h, while no substantial difference was observed for the Active-PBS-400 method at Re = 100 (8.41 ± 0.51 µm h^−1^) and the scratch assay (7.39 ± 0.28 µm h^−1^). 

In the chip with a side channel width of 400 µm, the increase in Re created a cell-free area with enhanced cell migration similar to that of the channel with a width of 200 µm (Figure 6G). As discussed in the previous section, increasing mechanical force on the cells during wounding hindered cell migration. In the Active-PBS-400 methods, the decrease in Re led to the cells being subjected to a higher mechanical force for a longer duration. Because, under lower Re, PBS streamed deeper into the side channel (Figure 5B), and more cells in the side channel were removed during wounding (296,672 µm^2^, 266,862 µm^2^, and 229,191 µm^2^ for the Active-PBS-400 method at Re = 25, 50, and 100, respectively, Figure 6G). Furthermore, in the device with a side channel width of 400, it was revealed that, in the Passive-Trypsin-400 method, there was the fastest migration due to chemical wounding with negligible mechanical force on the cells.

### 3.7. Comparison of All Methods of Chemical and Mechanical Woundings

**Figure 10:**
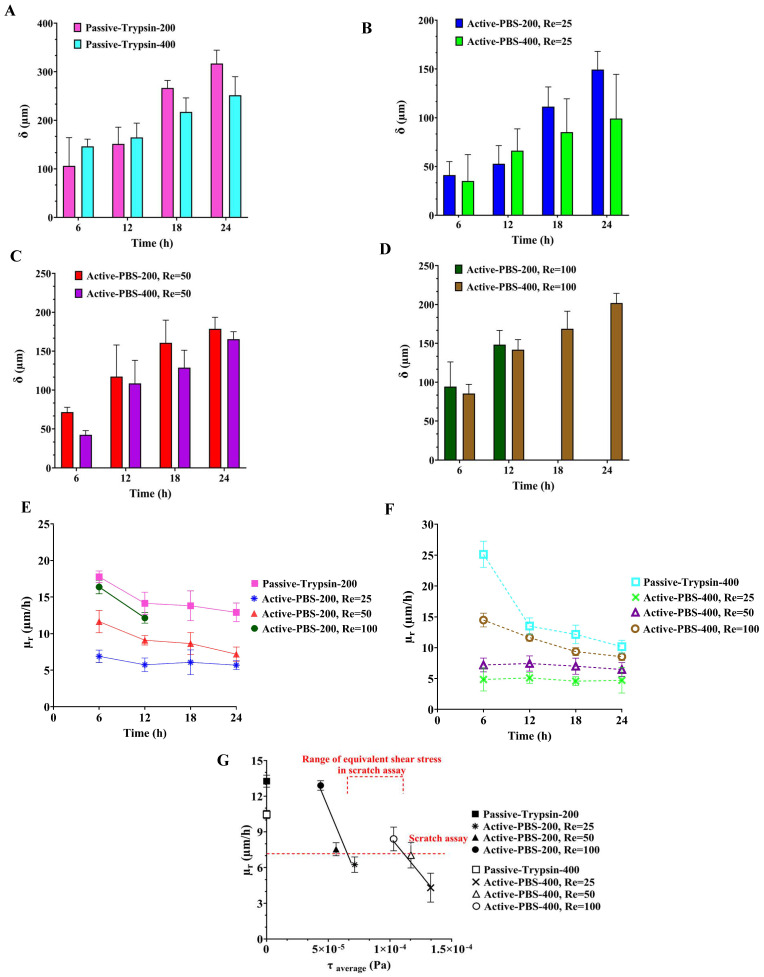
Comparison of cell migration by increasing the width of the side channel from 200 µm to 400 µm in four groups of (**A**) the Passive-Trypsin method and the Active-PBS methods at (**B**) Re = 25, (**C**) 50, and (**D**) 100. (**E**) Cell migration rate in chemical and mechanical microfluidic assays for the chip with side channel widths of (**E**) 200 µm and (**F**) 400 µm at 6, 12, 18, and 24 h after wounding. (**G**) Cell migration rate during migration with respect to the average shear stress subjected to the residual cells of the side channel in wounding to obtain a range of equivalent shear stress in the scratch assay.

In this section, first, the effect of increasing the side channel width from 200 to 400 µm on cell migration was compared in four groups of wounding, including the Passive-Trypsin method and the Active-PBS method at Re = 25, 50, and 100 (Figure 10A–D). In each wounding group, the time for cell-free creation was equal between the side channel widths of 200 and 400 µm. The results demonstrate that the increase in side channel width from 200 to 400 µm reduced cell migration (after 24 h, Passive-Trypsin-200: 316.91 ± 27.47 µm vs. Passive-Trypsin-400: 251.55 ± 38.25 µm (Figure 10A), Active-PBS-200 at Re = 25: 149.33 ± 18.7 µm vs. Active-PBS-400 at Re = 25: 114.26 ± 53.38 µm (Figure 10B), Active-PBS-200 at Re = 50: 178.68 ± 14.83 vs. Active-PBS-400 at Re = 50: 165.39 ± 9.6 µm (Figure 10C), and after 12 h, Active-PBS-200 at Re = 100: 148.34 ± 18.32 µm vs. Active-PBS-400 at Re = 100: 141.77 ± 12.90 (Figure 10D). The increase in the side channel width in each group resulted in more cells being exposed to mechanical forces during wounding (Figure 5A,B), leading to a more inhibitory effect on cell migration under high mechanical wounding. Therefore, increasing the side channel width could not help improve cell migration in the proposed microfluidic device due to increased mechanical shear force. 

The cell migration rate for chemical and mechanical microfluidic assays at each time point is shown in Figure 10E,F. It was found that the cell migration rate decreased over time and reached a constant tendency for cell migration. Figure 10E,F indicated that in mechanical microfluidic assays, the increase in Re and the decrease in the width of the side channel resulted in an increased migration rate closer to the chemical microfluidic assay (Passive-Trypsin-200), which had the highest migration rate. The increased Re and decreased side channel width in mechanical assays reduced mechanical stress on the cells, thereby mimicking the less invasive conditions of chemical assays and leading to higher migration rates. This shows that by optimizing flow conditions to minimize mechanical damage, mechanical microfluidic assays can achieve cell migration rates comparable to those seen in chemical assays, where enzymatic interactions are the primary driver. However, this increase in Re and decrease in the side channel width limited us to quantifying cell migration for fewer time points. For example, in Active-PBS-200 at Re = 100, the cell migration could be studied for 12 h as the cells had already migrated to the baseline.

The general process of cell-free creation in both the mechanical microfluidic assay and the scratch assay is mechanical. While the cell-free area was created with mechanical stress in the scratch assay, wounding was created with the shear stress of the fluid flow in the mechanical microfluidic migration assay. Figure 10G shows the migration rate during migration with respect to the average shear stress subjected to the residual cells of the side channel in wounding. The possible range of shear stress for the scratch assay can be determined where the migration rate of the scratch assay is the same as the mechanical microfluidic assays in Figure 10G. The shear stress range for the scratch assay is between 6.38 × 10^−5^ Pa and 1.10 × 10^−4^ Pa. Also, Figure 10G helps obtain our desired migration rate based on two factors: a side channel width and an average shear stress subjected to the residual cells in wounding by extrapolating or interpolating.

### 3.8. Comparsion of Cell Migration in Microfluidic and Scratch Assays 

By comparing cell migration in the microfluidic wound-healing assay with the scratch assay in the literature, Meer et al. [48] introduced a microfluidic wound-healing assay where the cell-free space was generated by a laminar trypsin flow made with a syringe pump. In their control condition, it was shown that the migration of human umbilical vein endothelial cells (HUVECs) in the scratch assay was two-fold higher than in the microfluidic assay. Also, a microfluidic wound-healing device was developed by Wei et al. [55] to characterize vascular smooth muscle cells (VSMSs) migration. Gravity as a driven force was utilized to create the cell-free area by laminar trypsin flow, similar to our method for the Passive-Trypsin method. It was found that in the control condition, cell migration in the scratch assay was enhanced almost 2.5 times more than in the microfluidic assay. However, in our microfluidic wound-healing assay, microglia BV2 migration in four cases (the Passive-Trypsin-200/400 methods and the Active-PBS-200/400 methods at Re = 100) was faster than the scratch assay. This discrepancy in results suggests that our microfluidic wound-healing migration assay may offer a more effective alternative to the scratch assay. Potential reasons for this discrepancy could include differences in cell types, assay conditions, or mechanical and chemical stimuli used in generating the wound. Further investigation into these factors could provide a deeper understanding of the underlying mechanisms affecting cell migration in various assay setups. 

## 4. Conclusions

In this study, we introduced a microfluidic device capable of quantifying cell adhesion and migration. In the control condition, microglia BV2 adhesion was characterized by assessing the cell detachment under a wide range of shear stresses. The device enabled us to study cell migration when the cell-free area was generated under chemical and mechanical stimuli with trypsin and PBS flow, respectively, compared to the scratch assay. In the chemical assay, gravity as a driving force was used to flow the trypsin within the chip to create the cell-free area without peripheral equipment. It was found that cell migration in the chemical wounding assay with trypsin was faster than in the wounding created mechanically with PBS flow and scratching. This observation suggests that the higher mechanical force acting on the cells during mechanical wounding may severely affect cell migration. Further studies are needed to confirm this potential mechanism. Our data presented that, as the wound was generated mechanically with the PBS flow, not only did the experiment time reduce, but cell migration was also enhanced by increasing inlet Re. Our study on the geometry of the device showed that the increase in the side channel width reduced cell migration. The privileges and bi-functionalities of the device made it a flexible and cost-effective assay to further a quantitative understanding of cell adhesion and migration.

## Figures and Tables

**Table 1:** Shear stress ranges in A_low_, A_medium_, and A_high_ at Re = 25, 50, and 100.

	Shear Stress in A_low_ (Pa)	Shear Stress in A_medium_ (Pa)	Shear Stress in A_high_ (Pa)
**Re = 25**	0–5	5–7	7–9
**Re = 50**	0–8	8–14.5	14.6–17
**Re = 100**	0–20	20–30	30–34

**Table 2:** Shear stress contours in A_low_, A_medium_, and A_high_ at Re = 25, 50, and 100.

	A_low_	A_medium_	A_high_
**Re = 25**	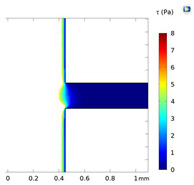	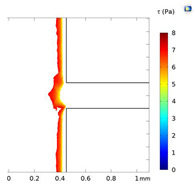	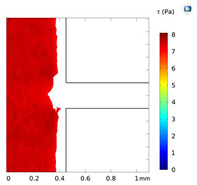
**Re = 50**	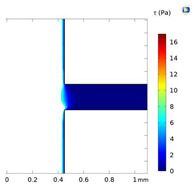	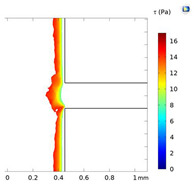	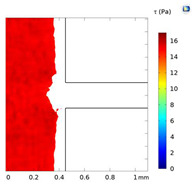
**Re = 100**	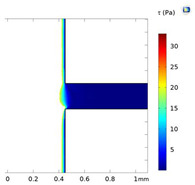	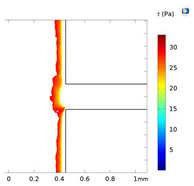	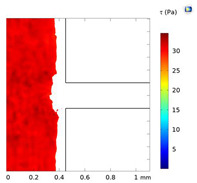

## Data Availability

The original contributions presented in the study are included in the article/Supplementary Materials, further inquiries can be directed to the corresponding author.

## Supplementary Materials

The following supporting information can be downloaded at: https://www.mdpi.com/article/10.3390/mi15081004/s1, Supplementary movie: discharging of the liquid within the microfluidic device using gravity.
